# Emergency in pediatric rheumatology: a narrative review

**DOI:** 10.3389/fmed.2026.1799500

**Published:** 2026-05-18

**Authors:** Francesca Casini, Luca Bernardo, Clelia Di Mari, Marta Brambilla, Lisa Gamalero, Martina Sandini, Sara Rotunno, Emanuele Bizzi, Angela Mauro, Teresa Giani

**Affiliations:** 1 Pediatric Unit, Department of Pediatrics, Vittore Buzzi Children’s Hospital, ASST Fatebenefratelli-Sacco, Milan, Italy; 2 Pediatric Unit, Department of Childhood and Developmental Medicine, ASST Fatebenefratelli-Sacco, Milan, Italy; 3 ULSS2 Marca Trevigiana, Conegliano Hospital, Conegliano, Italy; 4 Pediatric Rheumatology Unit, Department of Childhood and Developmental Medicine, ASST Fatebenefratelli-Sacco, Milan, Italy; 5 Ospedale San Pietro Fatebenefratelli, Rome, Italy; 6 Department of Internal Medicine, Vita-Salute San Raffaele University, Milan, Italy; 7 Rheumatology Unit, IRCSS, Meyer Children’s Hospital, Firenze, Italy

**Keywords:** children, emergency, pediatric, pediatric rheumatology, toxicity

## Abstract

Emergencies in pediatric rheumatology practice are relatively uncommon but rapid diagnosis and treatment are fundamental. Emergencies can be often misdiagnosed but prompt recognition and treatment are fundamental in order to reduce mortality and morbidity. This narrative review is a non-systematic analysis of the literature addressing the main emergencies in pediatric rheumatology. We aim to summarize the most relevant and frequent emergencies in pediatric rheumatology and provide practical guidance for the diagnosis and the management of emergency rheumatic disease in childhood.

## 1 Introduction

Emergencies in pediatric rheumatology practice are relatively uncommon but rapid diagnosis and treatment are fundamental. Several factors can complicate the assessment of children requiring urgent care, such as age-related limits in communication, frequent underreporting of pain, and short-term recall. Anxiety may hinder examinations and procedures, while physicians must also manage parental concerns, such as misperceptions of illness, denial of severity, or delayed care-seeking (1, 2). The purpose of this review is to summarize the most relevant and frequent emergencies in pediatric rheumatology and to provide practical guidance for the diagnosis and management of rheumatic emergencies in childhood. Topics are organized by pathology, and each emergency is further subdivided into epidemiology, clinical presentation, diagnosis, and therapy to allow for intuitive and rapid consultation.

## 2 Materials and methods

This narrative review was based on a structured literature search conducted in PubMed/MEDLINE, Embase, and Scopus. We considered articles published in English between January 1, 2010 and December 31, 2025.

The search strategy combined terms related to pediatric rheumatology with keywords referring to emergency or life-threatening conditions, along with disease-specific terms. The complete search strategies for each database are reported in Supplementary Table S1.

We included original studies, cohort and case–control studies, case series, clinical guidelines, consensus statements, and review articles considered particularly relevant to the topic. Case reports were taken into account only when they provided meaningful clinical insights into rare or severe pediatric rheumatologic emergencies.

Studies were excluded if they involved exclusively adult populations, addressed non-rheumatologic conditions, did not focus on emergency or life-threatening presentations, were not published in English, or represented duplicate records across databases.

All retrieved records were initially screened by title and abstract, followed by full-text evaluation of potentially relevant articles. The number of studies identified, screened, excluded, and ultimately included for each disease entity is summarized in Supplementary Table S2, while disease-specific PRISMA flow diagrams are provided in the Supplementary material.

Overall, approximately 1,350 records were identified across the three databases, in line with the initial estimate reported in the manuscript. A small number of landmark studies published before 2010 were also retained when they were considered essential to support key aspects of pathogenesis, diagnosis, or management and had not been superseded by more recent evidence.

## 3 Connective tissue disorders emergencies

Connective tissue disorders (CTDs) are a heterogeneous group of systemic inflammatory disease characterized by an immune dysregulation, associated with the production of autoantibodies and/or autoreactive T cells, leading to multi-organ damage (3). The main CTDs in children include childhood-onset systemic lupus erythematosus (cSLE), systemic sclerosis spectrum disorders (SSc), juvenile dermatomyositis (JDM), Sjögren’s syndrome (SjS), and mixed connective tissue disease (MCTD). cSLE is associated with more severe organ involvement (particularly renal and central nervous system manifestations), higher disease activity, and greater corticosteroid requirement compared with adult-onset SLE (4, 5). JDM represents the most common idiopathic inflammatory myopathy (IIM) in childhood. According to the most recent EULAR/ACR classification criteria, the diagnosis of JDM is based on a weighted scoring system that incorporates skin manifestations, age of disease onset, other clinical manifestations, measures of muscle weakness, laboratory parameters, and muscle biopsy findings (6, 7). Sclerodermiform syndromes (SS) are a group of diseases characterized by the presence of thickened, sclerotic skin lesions. SS include a localized form, usually limited to the skin or underlying tissues, and juvenile systemic sclerosis (jSSc) which is characterized by cutaneous and visceral involvement (8, 9). Herein, we aim to describe the main clinical emergencies associated with connective tissue disorders in childhood.

### 3.1 Catastrophic antiphospholipid syndrome

#### 3.1.1 Definition

Catastrophic antiphospholipid syndrome (CAPS) is a rare, life-threatening condition occurring in a small subset of patients with antiphospholipid syndrome (APS), and represents the most severe manifestation within the APS clinical spectrum CAPS is an extremely aggressive condition mediated by antiphospholipid antibodies (aPL), and is characterized by the rapid development of widespread thrombosis and microthrombosis affecting both small and large vessels, leading to multiorgan involvement and failure. The CAPS Task Force, in September 2019, confirmed the following classification criteria: involvement of three or more organs, systems, and/or tissues developing within less than 1 week; histopathological evidence of microvascular thrombosis in at least one organ; and persistent aPL positivity. When only three of the four criteria are fulfilled, the condition is classified as probable CAPS (10, 11).

#### 3.1.2 Epidemiology

APS has an estimated annual incidence of approximately 5 cases per 100,000 persons and a prevalence of 40–50 cases per 100,000 persons (12, 13). Approximately 3% of the cases occur in patients younger than 15 years, with a mean age of onset of 10.7 years and a female: male ratio of 1.2:1 (14, 15). In about half of the pediatric cases, APS is primary and is typically characterized by an earlier disease onset and greater involvement of arterial vessels. Secondary APS is most associated with Systemic Lupus Erythematosus (SLE) and lupus-like diseases (15). CAPS occurs in fewer than 1% of patients with APS, and approximately 90% of reported cases involve adults (16). In pediatric patients, the mean age at CAPS onset is 11.5 ± 4.6 years, with a female prevalence, and a high frequency (69%) of primary APS followed by SLE-associated APS (29%) (17). CAPS in children often represents the presenting manifestation of APS and infections are the most frequent precipitating factors, followed by malignancies and disease flare of underlying autoimmune conditions (16).

#### 3.1.3 Clinical manifestation

The clinical presentation depends on the organs and tissues affected by thrombosis; renal, pulmonary, cerebral, hepatic, and cutaneous districts are the most frequently involved (10). Pediatric patients exhibit a similar clinical phenotype as adults, although with a higher prevalence of thrombotic events involving the peripheral vasculature (16). A rapidly progressive multiorgan functional deterioration especially in the presence of microangiopathic hemolytic anemia and thrombocytopenia, is highly suggestive of CAPS.

#### 3.1.4 Diagnosis

Several diseases may mimic CAPS, making the diagnosis particularly challenging. This includes sepsis, disseminated intravascular coagulation (DIC), thrombotic thrombocytopenic purpura (TTP), and hemolytic uremic syndrome (HUS). Common laboratory findings include microangiopathic hemolytic anemia, leukocytosis, elevated ferritin levels, increased inflammatory markers, and evidence of organ dysfunction, such as elevated cardiac biomarkers, creatinine, liver enzymes, and lactate dehydrogenase (LDH), as well as proteinuria, low haptoglobin levels, and hypocomplementemia. CAPS is considered part of the spectrum of hyperferritinemic syndromes, and ferritin appears to play not only a marker role but also active contribution to the disease pathogenesis (18). Laboratory signs of coagulation activation (thrombocytopenia, increased D-dimer and fibrinogen levels, prolonged blood coagulation markers) are frequently observed, in particular, activated partial thromboplastin time (aPTT) prolongation may suggest the presence of lupus anticoagulant may suggest the presence of lupus anticoagulant. The identification of aPL is crucial for the diagnosis. Current classification criteria include three aPL subtypes: lupus anticoagulant, anticardiolipin antibodies, and anti-β2GPI antibodies. These antibodies are usually detected in high titers, however lower titers may occasionally be observed due to antibody consumption during extensive thrombosis (19). Additionally, interpretation of aPL testing may be further complicated by concurrent treatments, such as anticoagulant therapy, or by intercurrent infections, which frequently act as CAPS triggers. The assessments should include assessment for infections, underlying autoimmune and additional prothrombotic conditions, including factor V Leiden and prothrombin (factor II) mutation, protein C and protein S deficiencies, or hyperhomocysteinemia.

#### 3.1.5 Treatment

Due to the rarity of this condition, no official treatment guidelines or randomized controlled trial (RCT)-level evidence are currently available. Although identification of triggering factors and potential secondary causes may allow for a more targeted treatment, the severity and the rapid progression of CAPS often require the prompt initiation of treatment, even before the definitive identification of the precipitating causes. The therapeutic strategy is based on three main objectives: supportive care, prevention and treatment of thrombotic events and suppression of the inflammatory response (20). Supportive treatment depends on the organs involved, and the empirical broad-spectrum antibiotic treatment should be evaluated (21). Anticoagulation represents a cornerstone of treatment. The choice of anticoagulant may include unfractionated heparin (UFH) or Low Molecular Weight Heparin (LMWH) (22). UFH can be administered intravenously or subcutaneously, with dose adjustments based on aPTT values. Its anticoagulant effect is rapidly reversible with protamine sulfate, making it particularly suitable for acute hospital settings. LMWH, such as enoxaparin or dalteparin, are administered subcutaneously, and provide a predictable and prolonged anticoagulant effect; with reversal is more challenging, and dose adjustments are monitored usinganti-Xa levels. The role of new oral anticoagulants in this condition is still unclear and controversial (23). Corticosteroids constitute a fundamental treatment in CAPS, although their effectiveness is difficult to assess, given that available data often show their use in conjunction with anticoagulant therapy (22). There are no specific recommendations regarding the type of corticosteroid or dosing; however, given the life-threatening nature of CAPS, high-dose intravenous methylprednisolone pulses are commonly administered (24). The coexistence of a severe infection may induce the use of lower intravenous corticosteroids doses. Improved outcomes have been observed with a combination treatment regimen including corticosteroids, heparin, and either plasmapheresis or intravenous immunoglobulin (IVIG) (19, 24–26). In some cases, IVIG treatment may be followed by plasma exchange. IVIG are usually administered at a dose of 0.4 g/kg/day for 5 days or 1 g/kg/day for 2 days (27). In patients with microangiopathic hemolytic anemia or renal dysfunction, plasmapheresis, especially when using fresh frozen plasma as the replacement fluid instead of albumin, might be preferred over IVIG in order to restore coagulation and complement systems (24, 28). The potential renal and vascular complications associated with IVIG should be carefully evaluated. IVIG preparations with low sucrose content, which is nephrotoxic, are preferred, and the combination with anticoagulant treatment needs to be ensured (24, 27). Immunosuppressive agents may be added to the above treatments in selected cases. Among these, cyclophosphamide (intravenous 500–750 mg/m2) and/or rituximab (intravenous 375 mg/m2 once weekly for 4 weeks, or 500 to 1,000 mg given twice, separated by 7 or 14 days) (29, 30). In the next future, drugs such as eculizumab (complement protein C5 inhibitor), sirolimus (mTOR inhibitor), belimumab (BAFF/Blys inhibitor), daratumumab (anti-CD38 monoclonal antibody), defibrotide (a heterogeneous mixture of polyanionic), or obinutuzumab (Type II anti-CD20 monoclonal antibody) could represent additional options for patients refractory to the classic triple therapy (31–36). Despite aggressive and timely treatment, CAPS remains associated with high mortality rates, exceeding 30% in adults and approximately 25% in pediatric patients. CAPS secondary to systemic lupus erythematosus is associated with the poorest outcomes (16).

### 3.2 Renal crisis

#### 3.2.1 Definition

Renal crisis is a rare but life-threatening complication of systemic sclerosis (SSc) characterized by malignant hypertension and acute kidney injury (AKI). In 2015 the Scleroderma Clinical Trials Consortium (SCTC) Working Group on Scleroderma Renal Crisis (SRC) was created with the aim of developing and validating classification criteria for renal crisis in SS (Table 1) (37). Steen et al. (38) proposed a framework for the pathophysiology of SRC consisting of a sequence of renal insults. An initial vascular injury, with consequent rapid platelet adhesion and aggregation, leads to narrowing or total obliteration of vessel lumen, resulting in reduced renal perfusion and tissue ischemia. Subsequent activation of the renin-angiotensin system leads to elevation in blood pressure. This contributes to further vasoconstriction and sets up a vicious cycle of hypoperfusion and ischemia that leads to kidney injury (38).

**Table 1:** Core set of 13 items in five domains to develop classification criteria for SRC (37).

Domain	Item
Blood Pressure (BP)	Acute rise in BP defined as any of the following: Systolic BP ≥ 140 mmHg Diastolic BP > 90 mmHg A rise in systolic BP ≥ 30 above normal A rise in diastolic BP > 20 above normal
Kidney injury	Acute kidney injury defined as any of the following: Increase in serum creatinine by >0.3 mg/dL (26.5 umol/l) within 48 h Increase in serum creatinine to ≥1.5-time baseline occurred within the prior 7 days Urine volume <0.5 mL/Kg/h for 6 h
Microangiopathic hemolytic anemia and thrombocytopenia	New or worsening anemia not due to other causes Schistocyte or others red blood cell fragments on blood smear Thrombocytopenia (platelet count ≤ 100.000/ul) Laboratory evidence of hemolysis (elevated lactate dehydrogenase, reticulocytosis, or low/absent haptoglobin) A negative direct anti-globulin test
Target organ dysfunction (not attributable to other causes)	Hypertensive retinopathy (hemorrhages, hard and soft exudates or disk edema) Hypertensive encephalopathy (headache, altered mental status, seizures, visual disturbances or focal/diffuse neurologic signs) Acute heart failure (breathless, ankle swelling, fatigue, elevated jugular venous pressure, pulmonary crackles or peripheral edema) Acute pericarditis, diagnosed with at least two of the following criteria; pericarditis chest pain, pericardial rub, new widespread ST-elevation or PR depression on electrocardiogram, pericardial effusion on cardiac echocardiography
Renal histopathology	Small vessel (arcuate and interlobular arteries) changes that predominate over glomerular alterations Glomerular changes of thrombotic microangiopathy. Since none of these findings are specific for SRC, the pathological diagnosis must be supported by appropriate clinical and serological data.

#### 3.2.2 Epidemiology

Renal crises in pediatric patients are extremely rare (39). Thus, the greater part of data available has been obtained from studies on adults, where the incidence of SRC rises to 20% of SSc patients. SRC is more common in patients with early (less than 5 years) and rapidly progressive diffuse cutaneous SSc as compared to limited cutaneous SSc. Other risk factors include anti-RNA polymerase III antibodies, tendon friction rubs, large joint contractures, recent anemia, cardiac events (e.g., heart failure and pericardial effusion, systemic arterial hypertension), proteinuria and chronic kidney disease (40, 41). A significant relationship between the use of high- dose steroids and the development of SCR has been reported. However, it is not clearly understood if it is causal or instead confounded by disease severity and/or possibly nephrotoxic co-interventions (42). A genetic susceptibility for SRC has been hypothesized (43, 44). In adults, 1-year outcomes remain poor, with over 20% mortality at 6 months, 30–36% at 1 year and almost 50% at 10 years from SRC onset. Renal recovery is reported in 25% of patients up to 18 months after SRC. The rate of permanent dialysis after SRC ranged from 19 to 40% (45, 46).

#### 3.2.3 Clinical manifestation

Renal crisis is characterized by a wide spectrum of symptoms that rises from moderate compromission of kidney function (hematuria, isolated proteinuria, increased level of creatinine and uremia) to AKI and from mild elevation of blood pressure (or even normotensive) to malignant hypertension (47). Microangiopathic hemolytic anemia occurs in up to 60% (48). Although data are limited, available evidence suggests that children may have better outcomes than adults in this regard (49).

#### 3.2.4 Diagnosis

The diagnostic work-up of SRC includes laboratory tests, cardiac echocardiogram and ophthalmoscopic exam (Table 1). Although kidney biopsy may be useful prognostically, its use must be balanced with the risks of bleeding in the setting of uncontrolled hypertension, anemia and thrombocytopenia. Thus, it is usually reserved for patients with atypical presentations (50).

#### 3.2.5 Treatment

In terms of preventive measures, regular blood pressure monitoring and seeking early attention when it suddenly increases are mandatory. Patients at high risk for SRC may benefit from shorter follow-up intervals with particular focus on subtle changes or abnormalities in proteinuria and glomerular filtration rate (GFR), intensive home blood monitoring (including 24-h ambulatory blood pressure monitors) and earlier nephrology consultation (51). No evidence has been published supporting the preventing use of angiotensin-converting enzyme inhibitors (ACEi) to decrease risk of development or improve outcome of SCR (52). If SRC is suspected, the patient must be admitted to the hospital. The main aims of treatment are to reduce malignant hypertension and restore the patient’s baseline blood pressure over 72 h (3–5 days), avoiding an excessively rapid reduction of blood pressure that can cause hypoperfusion of vital organs. ACEi should be immediately started. A long-acting agent (e.g., ramipril) is preferable in most cases as it can be easily up titrated to maximum dose, normally by doubling the dose at 24-h intervals. Short-acting agents (e.g., captopril) may be suitable in cases of hemodynamic compromise due to its rapid onset of action and shorter half-life with possibility of rapid dose escalation as necessary (48). Renal function can continue to deteriorate despite initiation of ACEi and correction of blood pressure. This should not prompt discontinuation of therapy. Additional agents can be added to achieve BP control, including calcium channel blockers and alpha antagonists (53). Doses and modality of administration are reported in Table 2. Angiotensin receptor blockers have not been proven to be as beneficial as ACEi because they do not inhibit the degradation of bradykinin, which are potent vasodilators. Beta blockers are generally avoided due to the potential for further vasoconstriction (48). Emerging reports describe the use of complement inhibitors in severe cases of scleroderma renal crisis associated with complement activation and clear evidence of microangiopathic hemolytic anemia. Eculizumab is a humanized recombinant monoclonal antibody, administered intravenously, directed against complement component 5 thus inhibiting the generation of C5a and C5b-9 and consequently the lysis and the endothelial damage. The rationale for its usage stands in the association of hypocomplementemia with SSc and vascular involvement, occurrence of microangiopathic hemolytic anemia in SRC, C5b-9 deposits in capillaries of SSc patients’ skin biopsies and C4d deposits in renal peritubular capillaries of SSc patients with a poor renal outcome (51). Plasma exchange has not demonstrated efficacy and should not be recommended, with the exception of rare SRC patients who might develop thrombotic microangiopathy associated with anti-ADAMTS-13 antibodies (51). In cases of irreversible renal failure or uncontrollable hypertension, some success has followed the use of hemodialysis with or without bilateral nephrectomy and transplantation (48, 54).

**Table 2:** Treatment of SRC (54).

Drug	Class	Dose	Dose interval	Route
Ramipril	ACEi	1.6 mg/m^2^/day up to 6 mg/m^2^/day	Daily	Oral
Captopril	ACEi	0.5 mg/kg per dose up to 6 mg/kg per dose	Three times a day	Oral
Amlodipine	Calcium channel blockers	1–5 y 0.1 mg/kg/day up to 0.6 mg/kg/day (up to 5 mg per d) ≥6 y 2.5 to 10 mg	Daily	Oral
Nicardipine	Calcium channel blockers	Bolus 30 mcg/kg up to 2 mg per dose Infusion 0.5–4 mcg/kg per min		Intravenous
Clonidine	Alpha antagonists	2–5 mcg/kg per dose up to 10 mcg/kg per dose	Three-four times a day	Oral

### 3.3 Capillary leak syndrome

#### 3.3.1 Definition

The term capillary leak syndrome (CLS) refers to a constellation of clinical manifestations associated with increased capillary permeability to proteins, including diffuse pitting edema, exudative serous cavity effusions, noncardiogenic pulmonary edema, hypotension, and hypovolemic shock leading to multiorgan failure. The pathogenesis of CLS remains unclear; however, it is characterized by an acute and intense inflammatory process resulting in increased capillary permeability. Mortality associated with CLS is high, with reported lethality rates of 20–30% in acute forms among adults (55). No data are available for the pediatric population. A wide range of conditions can give rise to capillary leak syndrome (CLS), including sepsis (the most common cause), infections (predominantly viral), autoimmune diseases (Kawasaki disease, antiphospholipid syndrome, SjS, systemic sclerosis, and polymyositis), ovarian hyperstimulation syndrome (OHSS), hemophagocytic lymphohistiocytosis (HLH), engraftment syndrome, viral hemorrhagic fevers (VHFs), differentiation syndrome, snakebite envenomation, ricin poisoning, and drug-related causes (including interleukins, monoclonal antibodies, and gemcitabine) (56). Among connective tissue diseases systemic lupus erythematosus, anti-phospholipid antibody syndrome and SjS have been associated with CLS flares. However, very little is known about the occurrence of capillary leak syndrome in these diseases.

#### 3.3.2 Epidemiology

The true incidence of CLS is unknown and varies from common to very rare, depending on the disease or drug in question. Indeed, many of the diseases leading to CLS are uncommon, and only a proportion of patients with a predisposing condition develop clinically significant CLS. Many cases of CLS are likely unrecognized and labeled as culture-negative sepsis or attributed to alternative pathophysiological mechanisms, such as hypoalbuminemia (56).

#### 3.3.3 Clinical manifestations

A typical acute episode of capillary leak syndrome (CLS) follows a triphasic course (57). The prodromal phase is characterized by normal capillary permeability and limited movement of fluid and proteins from the intravascular to the interstitial space (56, 58). Patients may present with nonspecific symptoms, such as deterioration of general condition (malaise, fatigue, weakness, dizziness, faintness, irritability) fever, digestive (abdominal pain, vomiting, and diarrhea) or flu-like symptoms (myalgia, cough, rhinorrhea). During the subsequent leak phase (1 to 2 days later), vascular permeability increases in response to cytokines, leading to the extravasation of protein-rich fluid into the interstitial space. The resulting intravascular volume depletion activates the renin–angiotensin–aldosterone system and the sympathetic nervous system, as well as vasopressin release (56). Sodium and water retention, together with massive accumulation of protein-rich fluid, lead to the development of generalized pitting edema, exudative serous cavity effusions, noncardiogenic pulmonary edema and/or acute respiratory distress syndrome, as well as gastrointestinal and muscle edema. Muscle edema may rarely progress to compartment syndrome or rhabdomyolysis. Moreover, the intravascular volume depletion also causes hypotension that can result in hypovolemic shock and multiple organ dysfunction syndrome. Hypovolemia and hemoconcentration are often complicated by deep vein thrombosis or pulmonary embolism and stroke. In the post-leak phase endothelial function is recovered, thus fluids shift back into the intravascular space. The BP normalized and edemas resolve through massive polyuria; however, organ failure can persist due to precedent hypoperfusion (56).

#### 3.3.4 Diagnosis

The diagnosis of CLS is challenging because of the nonspecific nature of its symptoms and should be considered in patients presenting with hypovolemia or unexplained shock associated with severe diffuse edema, hemoconcentration (median hematocrit value of 60.5%, with hemoglobin levels often exceeding 20 g/dL), and hypoalbuminemia (median value of 21 g/L) (55).

#### 3.3.5 Treatment

CLS is an exceedingly rare condition, randomized controlled trials are not feasible. Management is therefore largely empirical and based on clinical experience. Fluid management strategies represent the cornerstone of treatment. In patients with mild hypotension, intravenous boluses of isotonic crystalloids should be administered (10 mL/kg, up to a total of 40–60 mL/kg during the first hour of treatment) (59). In patients with severe shock, infusion of albumin (20% albumin, 0.5–1 g/kg administered over 3–4 h) represents a theoretically attractive resuscitative fluid for volume expansion. However, its effectiveness may be limited by ongoing loss of albumin from the intravascular compartment. To date, no clinical trials or case reports have evaluated the use of high–molecular-weight starches in pediatric patients (60). In patients who remain hypotensive, a trial of IV vasopressor therapy (norepinephrine 0.05–0.15 up to 3 μg/kg/min as first-line treatment) may be considered, although its effectiveness may be limited by intravascular volume depletion (59). Noncardiogenic pulmonary edema and worsening gut and muscle edema represent potential complications during fluid resuscitation, particularly after blood pressure stabilization, due to the shift of interstitial fluid back into the intravascular space. Therefore, a fluid-restrictive strategy, including reassessment of intravascular volume status and pulmonary function before further fluid administration, is essential. Once hemodynamic stability is achieved, loop diuretics (oral or intravenous furosemide, 1–3 mg/kg/day) should be instituted (60). Patients with stable BP and signs of fluid overload benefit from loop diuretic therapy. In those with borderline blood pressure and fluid overload, the combined administration of albumin and loop diuretics may represent an effective strategy for volume removal (60). Hormonal extravasation during leak phase (hypothyroidism, hypoadrenalism, hypogonadism, deficiency of hormone binding globulins, and hypogammaglobulinemia) provides a rationale for stress-dose steroid administration (IV methylprednisolone 30 mg/kg/day) and even thyroid hormone supplementation (55). Renal replacement therapy can be required in patients with AKI and oliguria (56). In patients with SCLS, polyvalent intravenous or subcutaneous immunoglobulins administration appears to be effective both as treatment (1–2 g Kg-1 daily) and as prophylaxis (1–2 g Kg-1 monthly) (61).

### 3.4 Congenital heart block

#### 3.4.1 Definition

Congenital heart block (CHB) is an atrioventricular (AV) conduction abnormality caused by the transplacental transfer of maternal anti-Ro/SSA, anti-La/SSB, or anti-U1-RNP antibodies, leading to immune-mediated injury and fibrosis of the fetal AV node and conduction system, resulting in first-, second-, or third-degree AV block (62). As reported by Vanoni et al. (63), these antibodies are typically found in mothers with systemic lupus erythematosus, SjS, rheumatoid arthritis, or undifferentiated connective tissue disease, though some women remain asymptomatic during pregnancy.

#### 3.4.2 Epidemiology

Worldwide, approximately 0.5–1% of women of reproductive age are positive for anti-Ro/SSA antibodies, and 1–2% of their pregnancy may be complicated by fetal cardiac abnormalities, particularly congenital heart block (CHB) of varying degrees (64–66). Third-degree complete CHB occurs in 2–4% of exposed fetuses and typically develops between 20 and 24 weeks of gestation. This condition has a mortality rate of 17–30% (67).

#### 3.4.3 Clinical manifestations

CHB typically develops between 18 and 24 weeks of gestation. The most frequent presentation is fetal bradycardia (heart rate <110 bpm), which may be detected during routine obstetric ultrasound or fetal echocardiography. Depending on the degree of block, affected fetuses may exhibit signs of hydrops fetalis, cardiomegaly, pericardial effusion, or heart failure (68). After birth, infants with complete AV block often present with persistent bradycardia, fatigue, feeding difficulty, cyanosis, or poor growth. Partial blocks may be asymptomatic but can progress over time (69, 70).

#### 3.4.4 Diagnosis

High maternal anti-Ro/SSA titers are strongly associated with an increased CHB risk. CHB may evolve along a spectrum from incomplete block, characterized by first-degree (CHB I) or second-degree (CHB II) atrioventricular (AV) delay, to complete or third-degree block (CHB III). This progression can be detected by progressive lengthening of the fetal AV interval, defined as a PR interval >3 SD above the mean or > 150 ms. While complete CHB is generally irreversible, isolated reports suggest that second-degree CHB may revert to sinus rhythm following prompt initiation of corticosteroid therapy (70). Evidence from the PR interval and dexamethasone evaluation (PRIDE) perspective study supports the use of serial fetal echocardiography to monitor the PR interval and detect early conduction delay in fetuses at risk for CHB (71). The transition from incomplete to complete CHB may occur in less than 24 h, defining a very narrow therapeutic window for effective intervention. Consequently, weekly echocardiography surveillance may miss this rapid progression, emphasizing the need for more frequent rhythm monitoring. Recent multicenter data indicate that home monitoring of fetal heart rate (FHR) by the mother, performed two to three times daily using a handheld Doppler device, may facilitate early detection of new-onset CHB II and allow timely initiation of therapy within this critical window (72, 73). This strategy increases the likelihood of identifying reversible conduction abnormalities before they progress to complete block.

#### 3.4.5 Treatment

Over the past two decades, physicians have attempted to identify CHB early and prevent its progression. In particular, several studies tried to screen positive anti-Ro/La pregnancies monitoring changes in the fetal heart that might precede CHB using fetal echocardiogram. However, the results of these screening and treatment efforts have produced contrasting results (74, 75). Steroid treatment has been suggested to prevent progression of CHB, although the effect is controversial. Prenatal treatment is indicated in fetuses with incomplete (first- or second-degree) blocks. The preferred regimen is dexamethasone 4 mg orally once daily (or equivalent fluorinated corticosteroid) for 2–4 weeks, continued if improvement or stabilization occurs. The goal is to reduce fetal inflammation and prevent progression to complete block (70). More recent data indicate treatment with hydroxychloroquine (HCQ) during pregnancy may be related to lower rates of CHB, suggesting that this drug could be used to prevent CHB. A practical regimen is HCQ 400 mg orally once daily, ideally initiated preconception or by ≤10 gestational weeks and continued throughout pregnancy. The PATCH trial prospectively showed that maternal HCQ reduced recurrent CHB by >50% compared with historical rates, using 400 mg/day started before completion of week 10 and maintained for the entire pregnancy (76, 77).

## 4 Emergencies in vasculitis

Vasculitis is a group of heterogeneous diseases, characterized by inflammation of the walls of blood vessels (78), defined by the Chapel Hill International Consensus of the Nomenclature of Systemic Vasculitis according to the vessels’ different size and type (79). The most common primary vasculitis in childhood is Henoch-Schönlein purpura (HSP), followed by Kawasaki disease (KD) (78). HSP is characterized by the presence of purpura (palpable and in crops) not related to thrombocytopenia (mandatory criterion) plus, at least, one of the following criteria: (1) acute onset diffuse colicky abdominal pain, (2) histopathology showing typically leucocytoclastic vasculitis with predominant IgA deposit or proliferative glomerulonephritis with predominant IgA deposit, (3) acute onset arthritis or arthralgias, and (4) renal involvement (80). KD is an acute, self-limiting febrile illness that predominantly affects children younger than 5 years of age. Diagnostic criteria for complete Kawasaki disease include fever lasting at least 5 days plus, at least, 4 of the following criteria: (1) cutaneous rash, (2) mucosal changes: lips erythema and cracking, oral and pharyngeal mucosa erythema, strawberry tongue, (3) extremities changes: hands and feet erythema and edema during the acute phase, periungueal desquamation during the subacute phase, (4) bilateral conjunctivitis without exudates, and (5) cervical lymphadenopathy, usually unilateral (81). Other vasculitis, even if less frequently, can affect children. Polyarteritis nodosa is characterized by the presence of histopathologic or angiographic abnormalities of small- or medium-size arteries (necrotizing vasculitis, aneurysm, stenosis, occlusion) plus at least one of the following criteria: (1) skin involvement, (2) muscular pain or tenderness, (3) systemic hypertension, (4) peripheral neuropathy, and (5) renal involvement (80). Deficiency of adenosine deaminase 2 is a genetic autoinflammatory disease characterized by the early development of a vasculopathy; it should be ruled out in case of polyarteritis nodosa with severe organ involvement and strokes (82). Takayasu arteritis can be diagnosed in case of angiographic abnormalities (mandatory criterion) and, at least, one of the following criteria: (1) pulse deficit or claudication, (2) discrepancy of four limb systemic blood pressure, (3) bruits over large arteries, (4) hypertension, and (5) increase in acute-phase reactants (80). Behçet disease involves patients younger than 16 years (pediatric Behçet disease) in 4 to 26% of cases. Three of the following six items are required to classify a patient as having pediatric Behçet disease: (1) recurrent oral aphthosis (at least three attacks/year); (2) genital ulcerations (typically with scar); (3) skin involvement (necrotic folliculitis, acneiform lesions, erythema nodosum); (4) ocular involvement (anterior uveitis, posterior uveitis, retinal vasculitis); (5) neurological signs (with the exception of isolated headaches); and (6) vascular signs (venous thrombosis, arterial thrombosis, arterial aneurysm) (83). Herein, we will describe the main clinical emergencies associated with vasculitis in children.

### 4.1 Alveolar hemorrhage

#### 4.1.1 Definition

Diffuse alveolar hemorrhage (DAH) is a severe, life-threatening syndrome caused by the leakage of blood from the pulmonary microvasculature into the alveolar spaces. It can derive from the damage of capillaries, arterioles, or venules lining the alveoli (84). The accumulation of blood in the alveoli creates a diffusion barrier, leading to hypoxemia, often progressing to acute respiratory distress syndrome (ARDS) (85). DAH can manifest as an isolated disorder, defined as idiopathic, or it can be secondary to multiple different etiologies (86–88). DAH stands out as one of the most severe and life-threatening manifestations of antineutrophil cytoplasmic antibody (ANCA)-associated vasculitis (AAV), occurring in up to 25% of adult patients, presenting as acute alveolar hemorrhage, chronic alveolar hemorrhage, or a combination of both. This group of vasculitides includes Wegener granulomatosis (WG), Churg-Strauss syndrome (CSS), and microscopic polyangiitis (MPA). In pediatric patients they are very rare, with an annual incidence that is estimated to be less than 1 case per one million children (89, 90). Diffuse chronic alveolar hemorrhage is part of the clinical spectrum of COPA syndrome as well as arthritis and renal disease. This rare monogenetic rheumatological disease is caused by mutations impacting a specific sequence of amino acids within the COPA gene, which encodes the COPα protein, and it has been recently suspected as linked to interferon (IFN) signaling pathway (91, 92). IgA vasculitis, commonly known as Henoch-Schönlein Purpura, is one of the most frequent vasculitis in children. Lung involvement has been rarely observed in the course of this disease where it has been described in a limited number of patients (93). These children described to develop lung involvement also had renal damage, suggesting a more severe disease course (94). Other infrequent immune-mediated conditions that can induce DAH in pediatric age are Behçet’s disease, Goodpasture syndrome, Evans syndrome, cryoglobulinemic vasculitis and juvenile idiopathic arthritis (95, 96).

#### 4.1.2 Epidemiology

Idiopathic pulmonary hemosiderosis is the most common cause of DAH in children, followed by infections, immune-mediated disorders, cardiac and vascular diseases (Table 3). Since it is a rare event, epidemiology is not well-defined in pediatric age. The incidence of the primary, idiopathic DAH has been estimated to range from 0.24 to 1.23 per million children per year, with significantly elevated numbers in those with trisomy 21, and with a median age at onset of 3 years (86, 97). Rheumatological diseases are among the most frequent secondary causes, and DAH can be their first, catastrophic, manifestation as reported in up to 20% of patients newly diagnosed during their intensive care unit admission (98). The median age at DAH onset in these patients is 4.5 years (86). Pulmonary hemorrhage is observed in 5 to 10% of pediatric SLE patients with lung involvement. It may manifest subtly as chronic low-grade hemorrhage with minimal clinical manifestations, but more frequently, it leads to an acute respiratory distress (99).

**Table 3:** Cause and management of DAH (86, 103, 104, 111).

Cause of DAH	Management
Pulmonary capillaritis	Pulse glucocorticoids + steroid-sparing immunosuppression + ev. Ig/plasmapheresis
Drug-induce	Withhold offending agent + supportive care
Diffuse alveolar damage	Treat underlying infection (if present) + supportive care, including lung-protective ventilation
Coagulopathy	Reverse coagulopathy+/−platelet transfusion
Pulmonary venous hypertension	Diuresis + cardiac medical optimization

#### 4.1.3 Clinical manifestations

DAH most common features include hemoptysis, dyspnea, chest pain, cough, weakness, and fatigue. Respiratory symptoms present as acute or even fulminant, but sometimes can be subtle and misleading, especially in the idiopathic forms (100). In a multicentric study involving 124 pediatric patients affected by DAH of any cause, 23% had no respiratory complaint (86).

#### 4.1.4 Diagnosis

Physical examination can reveal tachypnea, diffuse crackles, hemoptysis, sounds of consolidation, paleness, hypotension and hypoxemia. First-line laboratory tests, including hemoglobin levels, inflammatory markers, renal and hepatic function, hemogasanalysis, coagulation profile, complement fractions C3 and C4, as well as urine analysis (inclusive of proteinuria/creatininuria ratio, microscopic evaluation, and drug screening), can provide a first valuable guidance. Anemia is present in 87% of pediatric cases with DAH, serving as a reliable indicator of recent and ongoing bleeding and reflecting its severity (86). Patients without a clear etiology should further be investigated for infective causes and autoimmune serologies such as ANA profile, ANCA, Extractable nuclear antigens (ENAs), antiphospholipid antibodies, anti-GBM (glomerular basement membrane) antibodies, and immunoglobulins. The most common radiological finding are ground-glass opacities, patchy or diffuse infiltrates, consolidation, crazy-paving pattern, and atelectasis that can be detected at chest X-rays, and better defined at computed tomography (CT) and even more precisely at high-resolution computed tomography (HRCT) (101, 102). Broncoscopy with bronchoalveolar lavage (BAL) plays a crucial role in the diagnosis of DAH and in the identification of underlying conditions, and should be performed within the very first few days. BAL fluid can appear macroscopically bloody or hemorrhagic but sometimes siderophagic signs of alveolitis can be evidenced only in cytology (103, 104). This percentage aligns closely with the conventional Golde score, a semiquantitative method scoring the hemosiderin content in alveolar macrophages (105). In cases where the etiology is unclear or if there are multiple potential causes, a lung biopsy can help guide appropriate treatment strategies, although formal indications are not well defined (84). In summary, the “classic triad” of hemoptysis, drop in hemoglobin over 24–48 h, and new alveolar or interstitial infiltrates has a high suspicion for DAH.

#### 4.1.5 Treatment

Patients should promptly receive a supportive care to address impaired gas exchange (from oxygen supplementation to mechanical ventilation and even extracorporeal membrane oxygenation support), volume loss (through intravenous fluids and blood transfusions), and coagulation abnormalities (with fresh frozen plasma, or platelet transfusions, and/or vitamin K supplementation). Platelet counts are expected to be above the limit of 50,000/μL with a prothrombin time-international normalized ratio less than 1.5 (106). Prothrombotic treatments, including antifibrinolytics, especially lysine analogs like IV tranexamic acid, may also be considered (107). In those cases where an infection is identified or suspected, specific antimicrobial treatment is required; a broad-spectrum prophylactic treatment should also be considered in other forms of DAH. For immune-mediated DAH immunosuppressive therapy is the cornerstone. Methylprednisolone 1 to 2 mg/kg per day, intravenously or as high doses pulse therapy (e.g., 30 mg/kg per day up to 2 gram daily for 3–5 days) in severe cases or when a rapid response is needed is the starting treatment, then gradually tapered over the following 4 weeks (84, 108, 109). Once the patient stabilizes, oral prednisone is commonly used as a maintenance therapy (0.5–1 mg/kg per day, gradually tapered to the lowest effective dose to maintain control) (110, 111). After corticosteroids, IV cyclophosphamide (0.75 mg/m2) is the most commonly used drug and its association with corticosteroids increases patients’ survival (112). The successful use of one intravenous administration of Rituximab (RTX) (375 mg/m2 IV weekly for 4 weeks), a targeted antibody against the CD20 antigen on B-cells, has been described in a few case reports (113–115). The use of intravenous immunoglobulin (IVIG) of 2 g/kg/dose has been reported in pediatric cases with immune-mediated DAH in combination with immunosuppressive treatments and supportive care (110). In immune-mediated DAH, plasmapheresis may find a place allowing a rapid removal of circulating autoantibodies and immune complexes, that mediate the damage to the small pulmonary vessels. The American Society for Apheresis recommends plasmapheresis in patients with AAV and DAH who present with hypoxemic respiratory failure requiring either high-flow supplemental oxygen or mechanical ventilation (28) (Tables 3, 4). Nevertheless, while there is consensus regarding its definitive efficacy in Goodpasture’s syndrome, its effectiveness in patients with SLE remains a topic of controversy (116). Activated recombinant factor VII (FVIIa) is another potential treatment that can be administered both biomicroscopically or intravenously, with a rapid hemostatic effect (117). However, its use is reported in case series and case reports, while no official trials have tested its efficacy and safety. Patients with immune and nonimmune DAH had similar outcomes (118). Failure to diagnose and treat DAH syndromes in their early stages may lead to acute respiratory failure and death. Reported prognosis is poor, with in-hospital mortality ranging from 20 to 100% (119).

**Table 4:** Cause and management of pulmonary capillaritis (115, 116, 119).

Cause of pulmonary capillaritis	Management
Antiphospholipid antibody syndrome	Rituximab 375 mg/m^2^ IV weekly for 4 weeks
Anti-GBM antibody disease	Cyclosphamide 0.75 g/m^2^ IV AND plasma exchange
Systemic vasculitis	Cyclosphamide 0.75 g/m^2^ IV then initiation of oral maintenance dose 2 weeks later OR rituximab (dosing as above)
Systemic lupus erythematosis	Rituximab (dosing as above)

### 4.2 Gastrointestinal ischemia and bleeding

#### 4.2.1 Definition

Inflammation of the gastrointestinal vessels can lead to life-threatening conditions, with high morbidity and mortality rates despite advances in the management of vasculitis (78, 120). Mesenteric ischemia and massive bowel bleeding represent the most severe complications of mesenteric vasculitis (78, 120). Mesenteric ischemia consists of an interruption of the blood supply to a portion of the bowel resulting in bowel infarction and inflammatory changes (121). It represents a surgical emergency and may be caused by arterial or venous occlusion (embolism or thrombosis), bowel obstruction, or, in approximately 2% of cases, mesenteric vasculitis (122). In cases of intestinal ischemia occurring in children or young individuals, an underlying systemic disease should always be considered and ruled out (123). Massive gastrointestinal bleeding is a life-threatening emergency, resulting from various etiologies and requiring prompt and skilled resuscitation. Bleeding may originate from any segment of the gastrointestinal tract, including the small intestine, which cannot always be fully evaluated using conventional diagnostic techniques, thereby giving rise to the challenging clinical scenario of obscure massive gastrointestinal bleeding (124, 125).

#### 4.2.2 Epidemiology

Gastrointestinal involvement is common in pediatric vasculitis, whereas severe involvement is rare. Gendreau et al. (120) conducted a study on 213 patients (aged 14 to 94) with systemic vasculitis involving the gastrointestinal tract, to determine the rate of poor outcome, defined as admission to intensive care unit, emergency surgical intervention or death, and to highlight associated predictive factors. Poor outcomes were observed in 39% of the patients. The main risk factors identified were an underlying diagnosis of polyarteritis nodosa, poor performance status, morphine use, the presence of abdominal guarding, ileus, melena, leukocytosis, low hemoglobin levels, and elevated C-reactive protein (CRP) (120).

#### 4.2.3 Clinical manifestation

Clinical manifestations of intestinal ischemia are usually nonspecific and may include abdominal distension, tenderness, intestinal bleeding, and reduced bowel sounds. Symptoms can rapidly progress to peritonitis and shock (126). Gastrointestinal bleeding, depending on the site of origin, may present as hematemesis, melena, or hematochezia. In vasculitic disorders, bleeding is often multifocal and may coexist with ischemic or inflammatory lesions (124).

#### 4.2.4 Diagnosis

Laboratory findings such as elevated CRP, leukocytosis, metabolic acidosis and increased lactate can support the diagnosis of bowel ischemia or necrosis; nevertheless, abdominal x-rays and ultrasound are usually the first imaging studies performed in patients presenting with abdominal pain. These modalities can detect bowel obstruction, intussusception, or free air, but they have limited sensitivity for mesenteric ischemia. Nowadays, abdominal CT represents the first-line imaging test. Although not always present, characteristic CT features of bowel ischemia includes reduced or absent bowel wall enhancement and the presence of intramural gas. More frequent but nonspecific findings include bowel wall thickening, luminal dilatation, and intra-abdominal fluid. Catheter-based angiography represents the gold-standard for the diagnosis of intestinal ischemia, as it allows both diagnostic evaluation and therapeutic intervention; however, it is invasive and difficult to perform in pediatric patients. Exploratory laparotomy is required when there is a high clinical suspicion of ischemia for definitive diagnosis and treatment (126, 127).

#### 4.2.5 Treatment

The management of severe gastrointestinal complications is primarily based on hemodynamic stabilization, bowel rest, broad-spectrum antibiotics and surgical or angiographic intervention. Beyond this, the medical approach differs according to the different vasculitis.

##### 4.2.5.1 Henoch–Schönlein purpura (IgA vasculitis)

Gastrointestinal involvement is usually benign and self-limiting. Oral prednisone at a dose of 1–2 mg/kg/day for 2 weeks, followed by gradual tapering, is effective for moderate to severe abdominal pain, after ruling out intussusception, and protein-losing enteropathy. Its role in reducing the onset of complications has not been proven (128–130) (Table 5).

**Table 5:** Management of gastrointestinal emergencies in vasculitis (123, 131–135).

Henoch-Schonlein purpura Kawasaki disease Polyarteritis nodosa and deficiency of adenosine deaminase 2 Behcet disease	Treatment Prednisone 1–2 mg/kg/day PO for 2 weeks, then tapering ≥ in case of steroid-resistant vasculitis or disease relapsing: Mycophenolate mofetil: 300 mg/m^2^ IV, increased to 600 mg/m2 IV after 48 h, modified to 400 mg/m^2^ PO after 6 days, stopped after 32 weeks Cyclophosphamide: 500 mg/m^2^ IV single dose (administered with mesna) IV immunoglobulin: 2 g/kg over 1 day or 1 g/kg/dose for 2 days or 0.4 gr/kg/dose for 2 days Rituximab: 2 IV doses of 750 mg/m^2^ (max 1 gr) per dose, given 2 weeks apart IV immunoglobulin 2 g/kg over 10–12 h and aspirin 30–50 mg/kg/day until 48 h after defervescence, then 3–5 mg/kg/day as an antiplatelet dose High-dose corticosteroids (induction therapy): methylprednisolone 30 mg/kg/day IV for 3 days, then oral prednisone 1 mg/kg/day. Cyclophosphamide (induction therapy): 500–750 mg/m^2^ IV monthly for 6 months or fortnightly for 3 times followed by 2–4 monthly doses. Azathioprine (maintenance): oral 2 mg/kg/day. Mycophenolate mofetil (maintenance): oral 600 mg/m2 twice a day. ⇩IF NOT EFFICACY: Plasma exchange Methotrexate: 10–15 mg/m^2^/week orally or subcutaneously, Infliximab: 6 mg/kg IV at 0, 2, 4 weeks and then every 6 weeks Cyclosporine: 3–5 mg/kg orally twice a day Rituximab: 2 IV doses of 750 mg/m2 given 14 days apart Prednisolone 0.5–1 mg/kg per day for l–2 weeks, then tapered by 5 mg every week PO ≥ in case of steroid-resistant or steroid-dependent: Azathioprine/6-mercaptopurine: 2–2,5 mg/kg/day PO Thalidomide: 2 mg/kg/day PO Adalimumab: subcutaneously 160 mg at 0 w, 80 mg at 2 w, 40 mg at 4 w, then 40 mg s.c. every other week Infliximab: IV 5 mg/kg at week 0, 2, and 6 then every 8 weeks

For steroid-resistant or relapsing disease, case reports support:

- Mycophenolate mofetil: 300 mg/m2 intravenous (IV), increased to 600 mg/m2 IV after 48 h, modified to 400 mg/m2 per os (PO) after 6 days and discontinued after 32 weeks (131, 132).
- Cyclophosphamide: 500 mg/m^2^ IV single dose (administered with mesna) (133).
- Intravenous (IV) immunoglobulin: 2 g/kg over 1 day, or 1 g/kg/dose for 2 days, or 0.4 gr/kg/dose for 2 days (134).
- Rituximab: two IV doses of 750 mg/m2 (maximum 1 g per dose), administered two weeks apart (135).

Surgery is reserved for bowel infarction, perforation, or persistent intussusception.

##### 4.2.5.2 Kawasaki disease

Gastrointestinal manifestations generally improve with disease-specific therapy. The standard regimen includes IGV 2 g/kg over 10–12 h and oral aspirin 30–50 mg/kg/day until 48 h after defervescence, then 3–5 mg/kg/day as an antiplatelet therapy. When gastrointestinal symptoms precede classical features, misdiagnosis may lead to unnecessary surgery; therefore, Kawasaki disease should be suspected in febrile children with unexplained gastrointestinal inflammation (136, 137) (Table 5).

##### 4.2.5.3 Polyarteritis nodosa (PAN) and deficiency of adenosine deaminase 2 (DADA2)

Hemorrhage and intestinal ischemia are observed in one third of cases of adults with polyarteritis nodosa but are rare in children (138): in case of severe gastrointestinal involvement in pediatric PAN, deficiency of adenosine deamidase 2 (DADA2) should always be ruled out (78) (Table 5).

Treatment include:

- High-dose corticosteroids (induction therapy): methylprednisolone 30 mg/kg/day IV for 3 days, then oral prednisone 1 mg/kg/day.
- Cyclophosphamide (induction therapy): 500–750 mg/m^2^ IV monthly for 6 months or fortnightly for three doses, followed by 2–4 monthly doses.
- Azathioprine (maintenance): 2 mg/kg/day PO.
- Mycophenolate mofetil (maintenance): 600 mg/m2 twice a day PO.

Other treatments that can be used in case of inefficacy of the previous therapies are:

·Plasma exchange.
·Methotrexate: 10–15 mg/m2/week orally or subcutaneously
·Infliximab: 6 mg/kg IV at 0, 2, 4 weeks and then every 6 weeks.
·Cyclosporine: 3–5 mg/kg orally twice a day.
·Rituximab: two IV doses of 750 mg/m2 given 14 days apart (139, 140).

##### 4.2.5.4 Behçet disease

Management of intestinal Behçet disease (BD) generally parallels that of systemic BD and inflammatory bowel diseases (IBD) (141). In patients with severe symptoms or complications, induction treatment should include corticosteroids: oral prednisolone 0.5–1 mg/kg per day for l–2 weeks, followed by gradual tapering (5 mg every week) (Table 5).

Treatments that can be used in case of steroid-dependence or resistance include:

- Azathioprine /6-mercaptopurine: 2–2,5 mg/kg/day PO.
- Thalidomide 2 mg/kg/day PO.
- Adalimumab: SC 160 mg at week 0, 80 mg at week 2, 40 mg at week 4, then 40 mg s.c. every other week.
- Infliximab: 5 mg/kg IV at weeks 0, 2, and 6 then every 8 weeks (141, 142).

Surgical treatment is indicated in cases of bowel perforation, massive bleeding, or intestinal obstruction (141).

### 4.3 Kawasaki disease shock syndrome

#### 4.3.1 Definition

Kawasaki disease shock syndrome (KDSS) is a rare and severe form of Kawasaki disease (KD) characterized by hemodynamic instability with hypotension and signs of hypoperfusion (tachycardia, cool extremities, delayed capillary refill, oliguria, altered mental status). It involves mixed cardiogenic and distributive shock, secondary to systemic vasculitis, capillary leak, and myocardial dysfunction. In particular, the 2024 American Heart Association (AHA) Guidelines define KDSS as KD associated with systolic blood pressure ≤2 SD for age and sex or>20% fall from baseline, in the context of fever and diagnostic features of KD (143).

#### 4.3.2 Epidemiology

The incidence of KDSS is variable and it depends on geographic area. In fact, a lot of studies reported that the incidence of KDSS is higher in Western countries than in Asian regions. In this regard, Lin et al. compared KD patients with KDSS patients and they found that KDSS patients were older and more often developed coronary aneurysm (144).

#### 4.3.3 Clinical manifestations

KDSS is a multiorgan disease. Very often patients with KDSS meet criteria for incomplete KD and signs of cardiogenic and/or distributive shock appear at the onset of disease with fever <5 days (145). Regarding to KD criteria, KDSS presented a longer duration of fever and higher incidence of lymphadenopathy than KD patients but no difference in incidence were found in non-secretive conjunctivitis, oropharyngeal changes, cutaneous and perineal rash and extremity changes compared to KD. In addition to KD criteria, usually, patients with KDSS presented with gastrointestinal symptoms as acute surgical abdomen due to intestinal perforation /bleeding or colitis (136). Gámez-González et al. (146), analyzed the charts of 214 patients admitted to a tertiary pediatric center in Mexico City and reported that 5% of this cohort had KDSS and compared to KD patients, a higher percentage of patients presented abdominal signs and symptoms as abdominal pain, diarrhea and gastrointestinal bleeding (146). Patients with KDSS may show neurological symptoms including encephalopathy, cerebral hemorrhage and seizures due to cerebral vasculitis involving small and medium sized vessels (147, 148). Regarding cardiovascular involvement, typical of KD, pictures of myocarditis characterized by cardiac dilatation, reduced ejection fraction, areas of infarction and dyskinesia have been described in addition to aneurisms. Valvular abnormalities are also described due to valvulitis or papillary muscle dysfunction (149–151).

#### 4.3.4 Diagnosis

The early diagnosis of KDSS is crucial and is based exclusively on clinical features, since no specific diagnostic test is available. In the early stage, KDSS may closely mimic septic shock (SS) or toxic shock syndrome (TSS), leading to a potential delay in diagnosis and treatment. Park et al. retrospectively evaluated patients admitted with diagnoses of KD, KDSS, SS, and TSS to identify clinical and laboratory findings useful for a timely diagnosis of KDSS. They highlighted that patients with KDSS show a higher incidence of conjunctival injection, cervical lymphadenopathy, coronary dilations, and left ventricular dysfunction, along with markedly elevated inflammatory markers. Conversely, patients with TSS typically present with higher creatinine levels and positive serological or culture results (152). As in classical KD, KDSS patients often display significant elevations in inflammatory markers (CRP, ferritin), white blood cell and platelet counts, while severe inflammation may lead to reduced albumin and sodium levels due to persistent capillary leakage. Elevated transaminases and troponin I levels are also common, reflecting hepatocellular injury and myocardial involvement, respectively (153). Because KDSS is characterized by severe hyperinflammation, special attention should be paid to the possible development of macrophage activation syndrome (MAS)—a potentially life-threatening complication (154, 155).

#### 4.3.5 Treatment

The aim of these treatments is to reduce inflammation, decrease capillary leakage and reduce the risk of coronary artery abnormalities (156–158). Pharmacological approach of KDSS is reported in Table 6. Hemodynamic support (fluids, vasopressors) should be titrated to cardiac function (143).

**Table 6:** Management of KDSS according to AHA 2024 guideline (143).

First line of treatment	IVIG 2 g/kg infused over 8–12 h. Prednisone 2 mg/kg/day (max 60 mg) PO divided every 8 h, tapered over 15 days after CRP normalization. Low-dose aspirin 3–5 mg/kg/day PO once daily after defervescence, continued 6–8 weeks.
Second line of treatment	Methylprednisolone 30 mg/kg/day (max 1 g) for 3–5 days, followed by oral prednisone taper. Infliximab 10 mg/kg IV once (preferred anti-TNF). Anakinra 2–10 mg/kg/day SC or IV divided every 12 h, discontinued when afebrile and improving. Cyclosporine 5 mg/kg/day PO in two doses, tapered by 10% every 3 days once CRP ≤ 1 mg/dL.
KDSS+MAS treatments	Add high-dose IV methylprednisolone pulses (30 mg/kg/day for 3–5 days) and either Cyclosporine 2–7 mg/kg/day or Anakinra up to 100 mg/day.

### 4.4 Stroke

#### 4.4.1 Definition

Stroke is defined as a prolonged or permanent alteration of brain function resulting from impaired cerebral blood flow (159).

Childhood stroke must be distinguished from perinatal stroke which is defined as cerebrovascular event occurring between 28 weeks’ gestation and 28 postnatal days of life with a pathogenesis usually related to maternal-fetal unit (160).

Pediatric stroke includes three subtypes:

- Ischemic stroke: caused by focal cerebral infarction due to arterial occlusion; it represents the most common type of pediatric stroke
- Hemorrhagic stroke: characterized by a spontaneous rupture of a cerebral vessel, most commonly associated with cerebral arteriovenous malformations.
- Cavernous sinus thrombosis: caused by deep venous thrombosis leading to venous infarction.

The etiology of childhood stroke includes genetic conditions such as cerebral vascular anomalies (particularly arteriovenous malformations), intrinsic abnormalities of blood vessels, and acquired factors such as encephalopathies and infections (161). Vasculitis may involve the cerebral nervous system (CNS) as a consequence of systemic inflammation and represents an important cause of pediatric stroke, which is commonly an ischemic stroke (162).

#### 4.4.2 Epidemiology

Stroke remains a leading cause of death and disability worldwide. However, pediatric-specific epidemiological data are limited, although several global epidemiology studies have recently improved the understanding of childhood stroke burden (163–165). Childhood stroke is primarily influenced by hereditary factors that increase susceptibility as well as by acquired triggering factors (166). The global incidence of stroke in children is estimated at approximately 2–13 cases per 100,000 children per year, which is lower than that reported in neonates (20–40 per 100,000 live births) and adults (80–90 per 100,000 person-years) (164, 167, 168). Large and medium vessel vasculitis, such as KD and Takayasu disease (TA) are more frequently associated with stroke than small vessel vasculitis. In KD, stroke has been described as a possible consequence of systemic inflammation and aneurysm formation, which may complicate the disease course, as well as a potential adverse effect of immunoglobulin therapy related to increased blood viscosity and immunoglobulin-induced arterial vasospasm (169–171). In most cases, stroke involves the middle cerebral artery territory, likely reflecting the predilection of KD for medium-sized arteries (172, 173). In TA, stroke has been reported in approximately 3.9 to 79% of patients, mainly due to large-vessel occlusion, and represents a significant cause of morbidity and mortality (174). In contrast, central nervous system involvement in small-vessel vasculitis is more frequent in ANCA-positive than in ANCA-negative subtypes (175) (see Table 7).

**Table 7:** Excluded criteria for IV tPA (176, 177).

EXCLUSION CRITERIA FOR IV tPA Unknown time of symptoms onset Pregnancy Clinical presentation suggestive of subarachnoid hemorrhage (SAH), even if brain imaging is negative for blood Patient who would decline blood transfusion if indicated History of prior intracranial hemorrhage Known cerebral arterial venous malformation, aneurysm or neoplasm Persistent systolic blood pressure more than 15% above the 95th percentile for age while sitting or supine Glucose less than 2.78 mmol/L or more than 22.22 mmol/L Bleeding diathesis including platelets less than 100,000, prothrombin time (PT) more than 15 s (international normalized ratio (INR) more than 1.4), or elevated activated partial thromboplastin time (aPTT) more than upper limits of the normal range Clinical presentation consistent with acute myocardial infarction (MI) or post-MI pericarditis that requires evaluation by cardiology before treatment Prior stroke, major head trauma, or intracranial surgery within the past 3 months Major surgery or parenchymal biopsy within 10 days (relative contraindication) Gastrointestinal or urinary bleeding within 21 days (relative contraindication) Arterial puncture at non-compressible site or LP within 7 days (relative contraindication). Patients who have had a cardiac catheterization via a compressible artery are not excluded. Patient with malignancy or within 1 month of completion of treatment for cancer. Patients with an underlying significant bleeding disorder. Patients with a mild platelet dysfunction, mild von Willebrand disease, or other mild bleeding disorders are not excluded. Stroke related exclusion criteria: Mild deficit (Paediatric National Institute of Health Stroke Scale (PedNIHSS) less than 4) at start of tPA infusion or at time of sedation for neuroimaging, if applicable. Severe deficit suggesting large territory stroke, with pre-tPA PedNIHSS more than 24, regardless of the infarct volume seen on neuroimaging. Stroke suspected to be because of subacute bacterial endocarditis, moyamoya, sickle cell disease, meningitis, bone marrow, air, or fat embolism. Previously diagnosed primary angiitis of the central nervous system (PACNS) or secondary central nervous system (CNS) vasculitis. Focal cerebral arteriopathy of childhood is not a contraindication. Neuroimaging related exclusions: Intracranial hemorrhage on pre-treatment head CT and MRI Intracranial dissection (defined as at or distal to the ophthalmic artery) Large infarct volume, defined by the finding of acute infarct on MRI involving one-third or more of the complete middle cerebral artery (MCA) territory involvement Drug-related exclusions: Known allergy to recombinant tissue plasminogen activator Patient who received heparin within 4 h must have activated partial thromboplastin time (aPTT) in normal range Low molecular-weight heparin (LMWH) within past 24 h (aPTT and INR will not reflect LMWH effect)

#### 4.4.3 Clinical manifestations

The clinical presentation of pediatric stroke is often more heterogeneous than in adults and is largely age dependent. Infants commonly present with nonspecific signs, such as seizures or altered consciousness, whereas older children more frequently exhibit focal neurological deficits (176). The clinical manifestations of childhood stroke vary according to stroke subtype and lesion extent. The most frequent symptoms include hemiparesis, hemifacial weakness, speech impairment, visual disturbances, and ataxia. Less commonly, children may present with nonspecific symptoms such as headache, nausea, and vomiting (177, 178).

#### 4.4.4 Diagnosis

The diagnosis of pediatric stroke may be challenging and is frequently delayed or missed, leading to worse outcomes. Both the American Stroke Association (ASA) and the Royal College of Paediatrics and Child Health (RCPCH) recommend the use of the FAST criteria (Face, Arms, Speech, Time) when stroke is suspected (179). However, the absence of FAST signs does not exclude the diagnosis of stroke (179). Acute assessment should include the age-appropriate Glasgow Coma Scale (GCS), considered pathological if <12, and/or the AVPU scale (Alert, Voice, Pain, Unresponsive), considered pathological if the patient responds only to voice at presentation (179). In case of suspected stroke, urgent hospital admission, possibly within an hour, is recommended.

The ABCD sequence (airways, breathing, circulation, disability) must be promptly evaluated and blood tests (including venous or capillary blood gas, coagulation, fibrinogen, urea, electrolytes, blood glucose, CRP, liver function tests and blood cultures) should be performed. Diffusion-weighted magnetic resonance imaging (MRI), or, if unavailable, non-contrast CT, combined with CT angiography (CTA) of the intra- and extracranial vessels in the presence of hemorrhage, or CTA of the neck and cranial vessels in the absence of hemorrhage, represents the first-line imaging assessment. CT angiography of the intra- and extracranial vessels is essential to evaluate the activity and extent of vasculitis, which may appear as diffuse vessel wall thickening and stenosis. If the primary imaging is negative but clinical suspicion remains high, a repeat MRI should be performed within 24 h. Regardless of the imaging modality, specialist interpretation and discussion with a reference neuroscience center are strongly recommended (179). The Pediatric National Institute of Health stroke Scale (PedNIHSS), derived from the analogous score in adults, may be used as a predictor of neurological deficit at 3 and 12 months. A PedNIHSS score equal or upper to 6 is considered significant (160).

#### 4.4.5 Treatment

The primary goal of acute treatment is to prevent further vascular injury and to preserve cerebral perfusion. In case of arterial ischemic stroke (AIS), RCPCH guidelines recommend initial treatment with aspirin (oral or nasogastric) at a dose of 5 mg/kg to a maximum of 300 mg within 24 h from the onset. After 14 days, the aspirin dose may be reduced to 1 mg/kg, up to a maximum of 75 mg. Tissue plasminogen activator (tPA) IV may be considered off label at the same dosage of adults (0.9 mg/kg) in children over than 2 years old within 4.5 h from the symptoms onset, in presence of PedNIHSS score between 4 and 24 and appropriate imaging criteria (177).

## 5 Juvenile idiopathic arthritis

Juvenile idiopathic arthritis (JIA) is an umbrella term referring to a heterogeneous group of inflammatory conditions that primarily affect the joints in children with disease onset before 16 years of age. It is defined by arthritis of unknown etiology persisting for more than 6 weeks. Extra-articular manifestations involving the eyes, skin, and internal organs may occur, potentially leading to significant disability and, in rare cases, life-threatening complications. JIA typically has an insidious onset with chronic joint inflammation affecting one or more joints. Limited range of motion and joint stiffness are the most common symptoms reported by patients and caregivers. Although any joint may be involved, cervical spine involvement is of particular concern, as it may lead to complications such as atlantoaxial subluxation.

In this section, we describe the main emergencies associated with JIA.

### 5.1 Anterior atlantoaxial subluxation and atlantoaxial impaction (AAI)

#### 5.1.1 Definition

Anterior atlantoaxial subluxation (aAAS) is a rare inflammatory complication, caused by an increase in the space between the arch of the atlas and the odontoid peg, subaxial subluxation (SAS) among the second and seventh cervical vertebrae, and erosions of the odontoid peg resulting in an “apple core” deformity (180–182). In a recent systematic review of the literature, an association with enthesis-related JIA subtype, high inflammatory markers and HLA B 27 positivity was noted in the patients with aAAS (183). Atlantoaxial impaction (AAI), or cranial settling, is another serious complication characterized by upward migration and/or rotation of the C2 complex (odontoid process) into the cranial vault due to C1-C2 instability. This might cause severe impinging of the brainstem or the craniovertebral junction, compression of the medulla and vertebral arteries (184).

#### 5.1.2 Epidemiology

The incidence of anterior atlantoaxial subluxation (AAS) in JIA is about 14% (183). Data regarding the prevalence of atlantoaxial impaction (AAI) in JIA are less precise. However, radiographic abnormalities of the cervical spine, including subluxation, joint inflammation, and ankylosis, are relatively common in children with JIA (185).

#### 5.1.3 Clinical manifestations

Patients may experience severe neck pain, stiffness, and signs of myelopathy. Moreover, airway management may be particularly challenging, and endotracheal intubation carries a high risk of odontoid process fracture in affected patients (180, 181, 185).

#### 5.1.4 Diagnosis

Diagnosis is clinically suspected based on symptoms and confirmed through imaging procedures as CT scan and MRI (186).

#### 5.1.5 Treatment

Treatment of cervical spine involvement includes medical, physical and surgical treatments. Oral non-steroidal anti-inflammatory drugs (NSAIDs) such as ibuprofen, naproxen, diclofenac, and indomethacin are used for symptomatic relief. Methotrexate (PO or SC) represents the first-line conventional disease-modifying antirheumatic drug (DMARD), while sulfasalazine or leflunomide (PO) may be considered in selected cases. In patients with persistent disease activity or axial involvement, biologic agents, particularly IV or SC anti–TNF inhibitors (etanercept, adalimumab, infliximab, golimumab) are commonly employed. Other biologics, including tocilizumab, abatacept, or rituximab, may be considered in refractory cases. In patients at high risk of cervical instability, cervical immobilization with a neck brace may be necessary. Surgical intervention is strongly recommended when signs of spinal cord compression are present, in order to stabilize the unstable cervical segment, decompress neural structures, and relieve pain related to mechanical instability (183, 187, 188) (Table 8).

**Table 8:** Treatment of atlantoaxial impaction (AAI) and anterior atlantoaxial subluxation (188).

Line of treatment	Drug class	Drugs	Pediatric dosage
First-line	NSAIDs	Ibuprofen (PO)	20–40 mg/kg/day in 3–4 doses
		Naproxen (PO)	10–20 mg/kg/day in 2 doses
		Diclofenac (PO)	2–3 mg/kg/day in 2–3 doses
		Indomethacin (PO)	2–3 mg/kg/day in 2–3 doses
		Meloxicam (PO)	0.125 mg/kg/day (max 7.5 mg/day)
Second-line	Conventional DMARDs	Methotrexate (SC or PO)	10–15 mg/m^2^/weeks
		Sulfasalazine (PO)	30–50 mg/kg/day in 2 doses
		Leflunomide (PO)	10–20 mg/day (weight-based)
	Biologic DMARDs	Etanercept (SC)	0.8 mg/kg/week (max 50 mg)
		Adalimumab (SC)	24 mg/m^2^ every 2 weeks (max 40 mg)
		Infliximab (IV)	5–6 mg/kg IV at weeks 0, 2, 6, then every 6–8 weeks
		Golimumab (SC)	30 mg/m^2^ monthly (max 50 mg)
		Tocilizumab (IV)	8–12 mg/kg IV every 2–4 weeks
		Abatacept (IV)	10 mg/kg IV at weeks 0, 2, 4 then monthly
Third-line	Surgical	Posterior cervical fusion/fixation	Indicated in instability or cord compression

## 6 Macrophage activation syndrome

### 6.1 Introduction

Macrophage activation syndrome (MAS) is a severe, potentially life-threatening hyperinflammatory condition classified as a form of secondary hemophagocytic lymphohistiocytosis associated with rheumatic and autoinflammatory diseases. It represents one of the most critical pediatric rheumatologic emergencies and requires prompt recognition and treatment to reduce mortality (189).

### 6.2 Epidemiology

MAS most commonly complicates sJIA, with an estimated incidence of 10–30%, but it may also occur in SLE, KD, juvenile dermatomyositis, SjS, and monogenic autoinflammatory disorders (190–195). Bennet et al., retrospectively analyzed data of patients with JIA and SLE complicated by MAS in a Pediatric Health Information System (PHIS) database using ICD-9-CM diagnosis codes for MAS and JIA or SLE, from 2006 to 2010. They enrolled 121 children at 28 children’s hospitals (102 with JIA and 19 with SLE). Among this cohort, patients at the first admission for MAS had JIA diagnosis much more common than SLE, and in particular sJIA (88% of patients). JIA patients were younger than SLE ones. No difference in ferritin levels were found between children with JIA and SLE but this level was checked earlier in JIA hospitalization than in children with SLE. Children with JIA had similar mortality compared to children with SLE and it was 7% (196).

### 6.3 Clinical manifestations

Clinically, MAS is characterized by persistent high fever (>38.5 °C) unresponsive to antipyretics, hepatosplenomegaly, lymphadenopathy, and rapid clinical deterioration. Neurological involvement (irritability, altered consciousness, seizures), hemorrhagic manifestations, respiratory distress, and multiorgan dysfunction may develop, particularly in severe or untreated cases (197).

### 6.4 Diagnosis

Laboratory abnormalities are central to diagnosis and include marked hyperferritinemia, typically with rapidly rising values, cytopenia (especially thrombocytopenia and leukopenia), hypofibrinogenemia, hypertriglyceridemia, elevated liver enzymes, and increased D-dimer levels. A characteristic feature is the paradoxical association of high CRP levels with low erythrocyte sedimentation rate, mainly due to fibrinogen consumption. Bone marrow hemophagocytosis, although considered a diagnostic hallmark, is detected in only up to 60% of patients and may be absent in early disease stages, limiting its diagnostic sensitivity (197). To support early recognition, several diagnostic and classification tools have been developed, including the HLH-2004 criteria, the 2016 EULAR/ACR/PRINTO classification criteria for MAS complicating sJIA, the MS score, the ferritin-to-erythrocyte sedimentation rate ratio, and composite scores differentiating MAS from primary HLH (198–201) (Table 9).

**Table 9:** Diagnostic/classification criteria of MAS (201–204, 219).

HLH 2004 (201)	2016 MAS classification criteria (202)	MS score (203)	Ferritin: ERS ratio (204)	MH score (219)
Molecular diagnosis	Fever in sJIA pt	Calculation ≥2.1 B-coefficient	Ferritin/erythrocyte sedimentation rate ≥ 21.5	(1) Points add to ≥60
or	AND		Platelets × (−0.003) + LDH × 0.0001 + fibrinogen × (−0.004) + ferritin × 0.0001	
5/8 Criteria:	Ferritin > 684 ng/mL	CNS 2.44		Age at onset (y) 0 (>1.6), 37 (≤1.6)
Fever > 38.5°C	AND	Hemorrhagic 1.54 Arthritis − 1.30		Neutrophils 0 (>1.4), 37 (≤1.4) (×10^9^/L)
Splenomegaly	2/4 Criteria:	Platelets (×10^9^/L) − 0.003		Fibrinogen (mg/dL) 0 (>131), 15 (≤131)
Cytopenia in 2–3 lines	Platelets ≤181 × 10^3^/mL			
Hemoglobin < 9 g/dL		LDH (U/L) 0.001		Splenomegaly 0 (no), 12 (yes)
Platelets < 100 × 10^3^/mL	AST > 48 u/L			
Neutrophil < 1 × 10^3^/mL	Triglycerides > 156 mg/dL	Fibrinogen (mg/dL) − 0.004		Platelets (×10^9^/L) 0 (>78), 11 (≤78)
Triglycerides ≥ 265 mg/dL and/or fibrinogen ≤ 150 mg/dL	Fibrinogen ≤ 360 mg/dL	Ferritin (ng/mL) 0.0001		Hemoglobin (g/dL) 0 (>8.3), 11 (≤8.3)
Hemophagocytosis				
Low natural killer cell				
Ferritin ≥ 500 ng/mL				
Elevated sIL-2 receptor				

### 6.5 Treatment

Early and aggressive treatment is essential. First-line therapy consists of high-dose intravenous corticosteroids, typically methylprednisolone 30 mg/kg/day (maximum 1 g/day) for 3–5 consecutive days, followed by oral prednisone at 2 mg/kg/day with gradual tapering based on clinical and laboratory response (202). In corticosteroid-refractory or severe cases, cyclosporine A is frequently added at doses ranging from 2 to 7 mg/kg/day, achieving rapid disease control. However, its use is limited by potential neurotoxicity and nephrotoxicity and by the lack of efficacy on the underlying inflammatory disease, with possible disease flares after discontinuation (203). Over the past decade, interleukin-1 blockade with anakinra has emerged as a key therapeutic option, particularly in refractory or life-threatening MAS. Subcutaneous anakinra is commonly administered at 1–2 mg/kg/day, with higher or divided doses used in severe cases. In critically ill patients or those with impaired subcutaneous absorption, intravenous anakinra has been successfully used, demonstrating rapid clinical improvement and a favorable safety profile (204–208). Phadke et al. describe their experience about 19 patients with various rheumatic diseases (sJIA, SLE, KD) complicated by MAS treated with intravenous anakinra. They showed that this treatment option is effective in critical MAS patients with a safe and well tolerated profile (209) (Table 10). Despite therapeutic advances, MAS remains associated with significant morbidity and mortality, highlighting the need for heightened clinical awareness, early diagnosis, and standardized treatment strategies (210).

**Table 10:** Treatment of MAS (202–209).

Line of treatment	Drug class	Drug	Pediatric dosage
First-line	Corticosteroids	Methylprednisolone (IV)	30 mg/kg/day (max 1 g/day) for 3–5 consecutive days
		Prednisone (PO)	2 mg/kg/day with gradual tapering
Second-line	Calcineurin inhibitor	Cyclosporine A (IV or PO)	2–7 mg/kg/day
Third-line/refractory MAS	IL-1 inhibitor	Anakinra (SC)	1–2 mg/kg/day (higher or divided doses in severe cases)
		Anakinra (IV)	1–2 mg/kg/day or higher doses in critically ill patients
Rescue therapy	Cytokine blockade	High-dose anakinra (IV)	Up to 8–10 mg/kg/day in life-threatening MAS (reported in case series)

## 7 Conclusion

Rheumatological emergencies in pediatrics are a heterogeneous group of rare but life-threatening events, characterized by a complicated immunopathological mechanism and rapid progression. Prompt recognizement is challenging due to the rarity of conditions, non-specific symptoms and other overlapping events. Moreover, most current treatments in pediatrics are obtained by studies on adults or observational cohorts. A timely diagnosis, rapid and adequate treatment and multidisciplinary support are fundamental in order to improve outcomes and reduce morbidity and mortality. The increasing knowledge of the immunological mechanisms related to emergencies will allow the development of targeted and tailored therapeutic approaches. Further multicenter studies will be necessary to establish evidence-based management strategies and improve outcomes in these uncommon but fatal conditions.

## Glossary

CTDs: connective tissue disorders
cSLE: childhood-onset systemic lupus erythematosus
SSc: systemic sclerosis spectrum disorders
JDM: juvenile dermatomyositis
SjS: Sjögren’s syndrome
MCTD: mixed connective tissue disease
IIM: idiopathic inflammatory myopathy
SS: sclerodermiform syndromes
ANA: antinuclear antibody
jSSC: juvenile systemic sclerosis
MSG: minor salivary gland
CAPS: catastrophic antiphospholipid syndrome
APS: antiphospholipid syndrome
aPL: antiphospholipid antibodies
DIC: disseminated intravascular coagulation
TTP: thrombotic thrombocytopenic purpura
HUS: hemolytic uremic syndrome
LDH: lactate dehydrogenase
aPPT: activated partial thromboplastin
RCT: randomized controlled trial
UFH: unfractioned heparin
LMWH: low molecular weight heparin
IVIG: intravenous immunoglobulin
AKI: acute kidney injury
SCTC: scleroderma clinical trials consortium
SRC: scleroderma renal crisis
GFR: glomerular filtration rate
ACEi: angiotensin-converting enzyme inhibitors
BP: blood pressure
CLS: capillary leak syndrome
OHSS: ovarian hyperstimulation syndrome
HLH: hemophagocytic lymphohistiocytosis
VHFs: viral hemorrhagic fevers
CHB: congenital heart block
AV: atrioventricular
CHB I: first-degree congenital heart block
CHB II: second-degree congenital heart block
CHB III: third-degree congenital heart block
PRIDE: PR interval and dexamethasone evaluation
FHR: fetal heart rate
HCQ: hydroxychloroquine
HSP: Henoch-Schönlein purpura
KD: Kawasaki disease
DAH: diffuse alveolar hemorrhage
ARDS: acute respiratory distress syndrome
ANCA: antineutrophil cytoplasmic antibody
AAV: ANCA-associated vasculitis
WG: Wegener granulomatosis
CCS: Churg-Strauss syndrome
MPA: microscopic polyangitis
INF: interferon
ENA: extractable nuclear antigens
anti-GBM: anti-glomerular basement membrane antibodies
CT: computed tomography
HRCT: high-resolution computed tomography
BAL: bronchoalveolar lavage
RTX: rituximab
FVIIa: activated recombinant factor VII
CRP: C-reactive protein
IV: intravenous
PO: per os
SC: subcutaneous
DADA2: Deficiency of adenosine deaminase 2
PAN: polyarteritis nodosa
BD: Behçet disease
IBD: inflammatory bowel disease
KDSS: Kawasaki disease shock syndrome
AHA: American Heart Association
SS: septic shock
TSS: toxic shock syndrome
MAS: macrophage activation syndrome
CNS: central nervous system
TA: Takayasu disease
ASA: American Stroke Association
RCPCH: Royal College of Paediatrics and Child Health
FAST: face, arms, speech, time
GCS: Glasgow Coma Scale
AVPU: Alert, vocal, pain, unresponsive
ABCD: airway, breathing, circulation, disability
MRI: magnetic-resonance imaging
CTA: CT angiography
PedNHISS: Pediatric National Institute of Health Stroke Scale
AIS: arterial ischemic stroke
tPA: tissue plasminogen activator
MCA: middle cerebral artery
JIA: juvenile idiopathic arthritis
AAI: Anterior atlantoaxial subluxation and atlantoaxial impaction
aAAS: anterior atlantoaxial subluxation
SAS: subaxial subluxation
NSAIDs: nonsteroidal anti-inflammatory drugs
DMARD: disease-modifying antirheumatic drugs
PHIS: Pediatric Health Information System
