# Supplementary material for: Emergency in pediatric rheumatology: a narrative review
Source: Front Med (Lausanne). 2026 May 18;13:1799500. doi: 10.3389/fmed.2026.1799500 (PMC13222824; doi:10.3389/fmed.2026.1799500)
Supplement: Supplementary file 1 [file Data_Sheet_1.DOCX]

# PRISMA 2020 Flow Diagram — Congenital Heart Block (CHB)

Identification

Records identified from databases

Pubmed (n = 186)

Embase (n= 241)

Scopus (n= 298)

Total (n= 725)

Records removed before screening:

Duplicate records removed

(n=212)

Screening

Records screened by title/abstract

(n=513)

Records excluded

(n=402)

Reasons:

-Not pediatric/fetal (n=121).

- Not rheumatological/autoimmune (n= 98)

- Not emergency/life-threatening (n=86)

- Irrelevant topic (n=97)

Eligibility

Full-text articles assessed

(n=111)

Full-text articles excluded Included

(n=86)

Studies included in qualitative synthesis

Reasons: (n=25)

-No CHB-specific data (n=28) Additional landmark pre-2010 studies retained

-Maternal Disease only (n=19) (n=4)

- Duplicate cohorts (n=12)

Low relevance case reports (n=17) Final references used

- Non English (n=10) (n=29)

# PRISMA 2020 Flow Diagram — Kawasaki Disease Shock Syndrome (KDSS)

Identification

Records identified from databases

Pubmed (n = 214)

Embase (n= 286)

Scopus (n= 331)

Total (n= 831)

Records removed before screening:

Duplicate records removed

(n=254)

Screening

Records screened by title/abstract

(n=577)

Records excluded

(n=454)

Reasons:

-Not pediatric/rheumatologic (n=116)

- Review/commentary without usable data (n= 65)

- Not emergency/life-threatening (n=132)

- Not specific/ irrelevant topic (n=141)

Eligibility

Full-text articles assessed

(n=123)

Full-text articles excluded Included

(n=98)

Studies included in qualitative synthesis

Reasons: (n=25)

-No disease-specific emergency data (n=31) Additional landmark pre-2010 studies retained

-Adult only/non pediatric population (n=18) (n=2)

- Duplicate/overlapping cohorts (n=16)

- Low relevance/ case reports (n=23) Final references used

- Non English (n=10) (n=27)

# PRISMA 2020 Flow Diagram — Macrophage Activation Syndrome (MAS)

Identification

Records identified from databases

Pubmed (n = 392)

Embase (n= 518)

Scopus (n= 604)

Total (n= 1514)

Records removed before screening:

Duplicate records removed

(n=471)

Screening

Records screened by title/abstract

(n=1043)

Records excluded

(n=846)

Reasons:

-Not pediatric/rheumatologic (n=214).

- Review/commentary without usable data (n= 164)

- Not emergency/life-threatening (n=181)

- Not specific/ irrelevant topic (n=287)

Eligibility

Full-text articles assessed

(n=197)

Full-text articles excluded Included

(n=151)

Studies included in qualitative synthesis

Reasons: (n=46)

-No disease-specific emergency data (n=49) Additional landmark pre-2010 studies retained

-Adult only/non pediatric population (n=38) (n=4)

- Duplicate/overlapping cohorts (n=27)

- Low relevance/ case reports (n=24) Final references used

- Non English (n=13) (n=50)

# PRISMA 2020 Flow Diagram — Gastrointestinal Ischemia and Bleeding

Identification

Records identified from databases

Pubmed (n = 278)

Embase (n= 352)

Scopus (n= 401)

Total (n= 1031)

Records removed before screening:

Duplicate records removed

(n=301)

Screening

Records screened by title/abstract

(n=730)

Records excluded

(n=561)

Reasons:

-Not pediatric/rheumatologic (n=142).

- No GI-related (n= 168)

- Not emergency/life-threatening (n=121)

- Irrelevant (n=130)

Eligibility

Full-text articles assessed

(n=169)

Full-text articles excluded Included

(n=129)

Studies included in qualitative synthesis

Reasons: (n=40)

-No disease-specific emergency data (n=41) Additional landmark pre-2010 studies retained

-Adult only/non pediatric population (n=26) (n=5)

- Duplicate/overlapping cohorts (n=19)

- Low relevance/ case reports (n=28) Final references used

- Non English (n=15) (n=45)

# PRISMA 2020 Flow Diagram — Catastrophic Antiphospholipid Syndrome (CAPS)

Identification

Records identified from databases

Pubmed (n = 120)

Embase (n= 160)

Scopus (n= 180)

Total (n= 460)

Records removed before screening:

Duplicate records removed

(n=110)

Screening

Records screened by title/abstract

(n=350)

Records excluded

(n=250)

Reasons:

-Not pediatric/rheumatologic (n=72).

- Non specific APS ithout CAPS data (n= 83)

- Not emergency/life-threatening (n=58)

- Irrelevant topic (n=37)

Eligibility

Full-text articles assessed

(n=100)

Full-text articles excluded Included

(n=75)

Studies included in qualitative synthesis

Reasons: (n=25)

-No disease-specific emergency data (n=24) Additional landmark pre-2010 studies retained

-Adult only/non pediatric population (n=19) (n=5)

- Duplicate/overlapping cohorts (n=12)

- Low relevance/ case reports (n=13) Final references used

- Non English (n=7) (n=30)

# PRISMA 2020 Flow Diagram — Scleroderma Renal Crisis (SRC)

Identification

Records identified from databases

Pubmed (n = 95)

Embase (n= 140)

Scopus (n= 160)

Total (n= 395)

Records removed before screening:

Duplicate records removed

(n=90)

Screening

Records screened by title/abstract

(n=305)

Records excluded

(n=210)

Reasons:

-Not pediatric/rheumatologic (n=57).

- Systemic Sclerosis without SRC data (n= 68)

- Not emergency/life-threatening (n=49)

- Irrelevant (n=36)

Eligibility

Full-text articles assessed

(n=95)

Full-text articles excluded Included

(n=65)

Studies included in qualitative synthesis

Reasons: (n=30)

-No disease-specific emergency data (n=19) Additional landmark pre-2010 studies retained

-Adult only/non pediatric population (n=18) (n=5)

- Duplicate/overlapping cohorts (n=10)

- Low relevance/ case reports (n=11) Final references used

- Non English (n=7) (n=35)

# PRISMA 2020 Flow Diagram — Capillary Leak Syndrome (CLS)

Identification

Records identified from databases

Pubmed (n = 80)

Embase (n= 110)

Scopus (n= 130)

Total (n= 320)

Records removed before screening:

Duplicate records removed

(n=70)

Screening

Records screened by title/abstract

(n=250)

Records excluded

(n=180)

Reasons:

-Not pediatric/rheumatologic (n=107)

- Not emergency/life-threatening (n=39)

- Irrelevant (n=34)

Eligibility

Full-text articles assessed

(n=70)

Full-text articles excluded Included

(n=50)

Studies included in qualitative synthesis

Reasons: (n=20)

-No disease-specific emergency data (n=14) Additional landmark pre-2010 studies retained

-Adult only/non pediatric population (n=12) (n=5)

- Duplicate/overlapping cohorts (n=9)

- Low relevance/ case reports (n=10) Final references used

- Non English (n=5) (n=25)

# PRISMA 2020 Flow Diagram — Diffuse Alveolar Hemorrhage (DAH)

Identification

Records identified from databases

Pubmed (n = 150)

Embase (n= 200)

Scopus (n= 230)

Total (n= 580)

Records removed before screening:

Duplicate records removed

(n=150)

Screening

Records screened by title/abstract

(n=430)

Records excluded

(n=310)

Reasons:

-Not pediatric/rheumatologic (n=83)

- Non immune /No Vasculitic DAH (n= 103)

- Not emergency/life-threatening (n=72)

- Irrelevant (n=52)

Eligibility

Full-text articles assessed

(n=120)

Full-text articles excluded Included

(n=85)

Studies included in qualitative synthesis

Reasons: (n=35)

-No disease-specific data (n=26) Additional landmark pre-2010 studies retained

-Adult only/non pediatric population (n=19) (n=5)

- Duplicate/overlapping cohorts (n=14)

- Low relevance/ case reports (n=17) Final references used

- Non English (n=9) (n=40)

# PRISMA 2020 Flow Diagram — Stroke

Identification

Records identified from databases

Pubmed (n = 210)

Embase (n= 260)

Scopus (n= 300)

Total (n= 770)

Records removed before screening:

Duplicate records removed

(n=200)

Screening

Records screened by title/abstract

(n=570)

Records excluded

(n=420)

Reasons:

-Not pediatric/rheumatologic (n=121).

- Non vasculitic stroke (n= 156)

- Not emergency/life-threatening (n=87)

- Irrelevant (n=56)

Eligibility

Full-text articles assessed

(n=150)

Full-text articles excluded Included

(n=105)

Studies included in qualitative synthesis

Reasons: (n=45)

-No disease-specific data (n=36) Additional landmark pre-2010 studies retained

-Adult only/non pediatric population (n=23) (n=5)

- Duplicate/overlapping cohorts (n=17)

- Low relevance (n=19). Final references used

- Non English (n=10) (n=50)

# PRISMA 2020 Flow Diagram — Atlantoaxial Subluxation in JIA

Identification

Records identified from databases

Pubmed (n = 75)

Embase (n= 100)

Scopus (n= 120)

Total (n= 295)

Records removed before screening:

Duplicate records removed

(n=80)

Screening

Records screened by title/abstract

(n=215)

Records excluded

(n=150)

Reasons:

-Not pediatric/rheumatologic (n=42).

- No -atlantoaxial complicatiom (n= 49)

- Not emergency/life-threatening (n=36)

-Irrelevant (n=23)

Eligibility

Full-text articles assessed

(n=65)

Full-text articles excluded Included

(n=45)

Studies included in qualitative synthesis

Reasons: (n=20)

-No disease-specific emergency data (n=13) Additional landmark pre-2010 studies retained

-Adult only/non pediatric population (n=9) (n=5)

- Duplicate/overlapping cohorts (n=8)

- Low relevance/ case reports (n=10) Final references used

- Non English (n=5) (n=25)
