# Supplementary material for: Emergency in pediatric rheumatology: a narrative review
Source: Front Med (Lausanne). 2026 May 18;13:1799500. doi: 10.3389/fmed.2026.1799500 (PMC13222824; doi:10.3389/fmed.2026.1799500)
Supplement: Supplementary file 2 [file Data_Sheet_2.DOCX]

# Supplementary Table S1. Complete search strategy

| **PubMed search strategy:** |
| --- |
| ("pediatric rheumatology"[Title/Abstract] OR "pediatric rheumatic diseases"[Title/Abstract] OR children[Title/Abstract] OR pediatric[Title/Abstract]) AND (emergency[Title/Abstract] OR emergencies[Title/Abstract] OR "life-threatening"[Title/Abstract] OR critical[Title/Abstract]) AND ("catastrophic antiphospholipid syndrome"[Title/Abstract] OR "antiphospholipid syndrome"[Title/Abstract] OR "macrophage activation syndrome"[Title/Abstract] OR "hemophagocytic lymphohistiocytosis"[Title/Abstract] OR "diffuse alveolar hemorrhage"[Title/Abstract] OR "Kawasaki disease shock syndrome"[Title/Abstract] OR "Kawasaki disease"[Title/Abstract] OR vasculitis[Title/Abstract] OR "IgA vasculitis"[Title/Abstract] OR "Henoch-Schönlein purpura"[Title/Abstract] OR "polyarteritis nodosa"[Title/Abstract] OR "Takayasu arteritis"[Title/Abstract] OR "Behçet disease"[Title/Abstract] OR "scleroderma renal crisis"[Title/Abstract] OR "systemic sclerosis"[Title/Abstract] OR "capillary leak syndrome"[Title/Abstract] OR "congenital heart block"[Title/Abstract] OR stroke[Title/Abstract] OR "juvenile idiopathic arthritis"[Title/Abstract] OR "atlantoaxial subluxation"[Title/Abstract] OR "gastrointestinal ischemia"[Title/Abstract] OR "gastrointestinal bleeding"[Title/Abstract]) Filters: English language; publication date from 1 January 2010 to 31 December 2025. |
| **Embase search strategy** |
| ('pediatric rheumatology':ti,ab OR 'pediatric rheumatic diseases':ti,ab OR child*:ti,ab OR pediatric*:ti,ab) AND ('emergency':ti,ab OR 'life threatening':ti,ab OR critical:ti,ab) AND (disease-specific terms as above) Limits: English; 2010–2025. |
| **Scopus search strategy:** |
| TITLE-ABS-KEY("pediatric rheumatology" OR "pediatric rheumatic diseases" OR children OR pediatric) AND TITLE-ABS-KEY(emergency OR emergencies OR "life-threatening" OR critical) AND TITLE-ABS-KEY(disease-specific terms as above) Limits: English; 2010–2025. |

**Supplementary Table S2. Literature screening process by disease entity:** Counts are reported separately for each disease entity based on a unified search strategy applied across PubMed, Embase, and Scopus. Duplicates were removed prior to screening. Screening was performed by title and abstract, followed by full-text assessment. Articles were excluded if they did not meet inclusion criteria. A limited number of landmark studies published before 2010 were retained when essential.

| **Condition** | PubMed | Embase | Scopus | Total identified | Duplicates removed | Screened | Excluded | Full-text assessed | Full-text excluded | Included studies | Landmark pre-2010 | Final references |
| --- | --- | --- | --- | --- | --- | --- | --- | --- | --- | --- | --- | --- |
| **CHB** | 186 | 241 | 298 | 725 | 212 | 513 | 402 | 111 | 86 | 25 | 4 | 29 |
| **KDSS** | 214 | 286 | 331 | 831 | 254 | 577 | 454 | 123 | 98 | 25 | 2 | 27 |
| **MAS** | 392 | 518 | 604 | 1514 | 471 | 1043 | 846 | 197 | 151 | 46 | 4 | 50 |
| **GI Ischemia/Bleeding** | 278 | 352 | 401 | 1031 | 301 | 730 | 561 | 169 | 129 | 40 | 5 | 45 |
| **CAPS** | 120 | 160 | 180 | 460 | 110 | 350 | 250 | 100 | 75 | 25 | 5 | 30 |
| **SRC** | 95 | 140 | 160 | 395 | 90 | 305 | 210 | 95 | 65 | 30 | 5 | 35 |
| **CLS** | 80 | 110 | 130 | 320 | 70 | 250 | 180 | 70 | 50 | 20 | 5 | 25 |
| **DAH** | 150 | 200 | 230 | 580 | 150 | 430 | 310 | 120 | 85 | 35 | 5 | 40 |
| **Stroke** | 210 | 260 | 300 | 770 | 200 | 570 | 420 | 150 | 105 | 45 | 5 | 50 |
| **JIA AAS** | 75 | 100 | 120 | 295 | 80 | 215 | 150 | 65 | 45 | 20 | 5 | 25 |
